# Quality of life among patients living with epilepsy attending the neurology clinic at kenyatta national hospital, Nairobi, Kenya: a comparative study

**DOI:** 10.1186/1477-7525-11-98

**Published:** 2013-06-18

**Authors:** Daniel WC Kinyanjui, Dammas M Kathuku, John M Mburu

**Affiliations:** 1 Department of Mental Health, School of Medicine, Moi University College of Health Sciences, PO Box 4606, Eldoret, 30100, Kenya; 2 Department of Psychiatry, College of Health Sciences, University of Nairobi, Nairobi, Kenya; 3 Department of Psychiatry, College of Health Sciences, University of Nairobi, Nairobi, Kenya

## Abstract

**Background:**

Most of the studies on epilepsy in Kenya and indeed the sub-Saharan region of Africa mainly focus on prevalence, psychiatric profile, and factors associated with increased seizure burden. This being the first Kenyan and sub-Saharan African study assessing quality of life among people living with epilepsy, it will identify their ‘intangible’ needs and enable evidence-based intervention that would ultimately lead to a comprehensive management and better outcome.

**Methods:**

*Design:* A cross-sectional comparative study, using the World Health Organization Quality of Life questionnaire, a socio-demographic questionnaire, seizure burden and characteristics, drug and treatment profile questionnaires and the Mini-Mental state examination, among PLWE and those accompanying them, herein referred to as the normal healthy controls, attending the neurology clinic at Kenyatta National Hospital, Nairobi.

*Setting and subjects:* Study was carried out between October 2006 and February 2007 at the neurology clinic in the hospital where three hundred consecutive subjects who satisfied the inclusion criteria and gave consent were recruited.

*Statistical tests used:* Descriptive statistics were used to compute means, standard deviations as well as frequencies. Significance of associations was tested using the Chi square test statistic (x^2^), an independent samples t-test, analysis of variance (ANOVA) and a step-wise (forward) regression analysis. A p < 0.05 was considered statistically significant.

**Results:**

The mean quality of life among people living with epilepsy (49.90%) was significantly (p < 0.01) lower than that of the normal controls (77.60%) accompanying them and significantly impaired as compared to the hypothesized mean of 75^±^2.5%. Factors significantly (p < 0.05) associated with impairment of quality of life in those living with epilepsy were a low level of education, higher seizure burden, low annual income, unemployment, unskilled employment, and living in a rural residence.

**Conclusions:**

The mean quality of life of people living with epilepsy at Kenyatta National Hospital was significantly impaired and lower than that of the normal controls accompanying them. A comprehensive epilepsy management program is recommended to address this problem and its associated risk factors for the people living with epilepsy in Kenya.

## Introduction

Adjustment to a chronic illness, such as epilepsy is not merely a function of the severity or duration of the disorder or associated treatment adverse outcomes. Response from the family and ‘significant others’ with associated imbalance between expectations and reality may be more menacing than the illness itself [1].

A majority of studies from this region mainly focus on psychiatry morbidity and factors associated with poor control of epilepsy. However Muinga [2] found a positive correlation between occupation (50.8% not gainfully employed) and psychopathology among persons living with epilepsy (PLWE) in Kenya. Another local study [3] reported that 63% of the unemployed PLWE were poorly controlled. There was therefore a possibility that either the psychopathology made it difficult for PLWE to keep a job or they tended to develop more neurotic symptoms if unemployed. In fact, the symptom profile checklist in the earlier study [2] showed that 94% suffered recurrent headaches, 89% were easily frightened and felt unhappy most of the time, 78% experienced feelings of worthlessness and were easily tired, 72% were worried and tired all the time, 61% experienced suicidal thoughts, while 17–44% had difficulty enjoying life, showed indecisiveness, cried more than usual, couldn’t think clearly, suffered poor appetites, subjectively reported inefficiency at work and had experiences of poor sleep.

One of the very few studies on this same subject in Africa, is a study that was carried out in Tunisia [4], using the short form survey (SF-36) questionnaire. In that study increased seizure severity and frequency appeared to be particularly problematic in impairment of quality of life (QOL), and unlike earlier studies in other settings that reported significantly lower scores in almost all 8 SF-36 subscales, only general health, mental health and social functioning were significantly lower among the Tunisian PLWE. Differences based on cultural, religious, family and social support were implicated as contributing to these findings.

The paucity of data on QOL among PLWE in Kenya and in the region as compared to our western counterparts clearly has serious implications on the possibility of achieving the objectives of the Global campaign against epilepsy [5]. This is despite literature repeatedly showing impaired QOL among PLWE in various settings [6-8] and the enormous opportunity created by this information for evidence based intervention [9,10]. Various studies have shown seizure burden [4,7,11], impairment in activities of daily living, pain and discomfort as among the common physical domain outcomes that impact negatively on the QOL of PLWE. Other studies have demonstrated impairment in the psychological [8,12,13] and social [14-17] domains of QOL. Elsewhere, objective and subjective stigma with associated high levels of anxiety and depression [9,18], impaired sexual activities [19], lower rates of marriage and employment [12] have been reported as some of the effects of epilepsy on QOL.

Quality of life in PLWE remains an important area of research and the assessment should not only focus on the evaluation of seizures but also other life domains such as cognitive, emotional, socio-occupational functioning, health perceptions and general satisfaction with life [20]. Recognition of this multi-dimensional nature of QOL has also been reflected in the three to six domains that usually comprise its assessment. In this study the physical health, psychological, social relationships and environmental domains were used in the assessment of QOL among PLWE.

## Methods

### Site

The study was carried out at the adult neurology clinic, Kenyatta National Hospital (K.N.H), in Nairobi, Kenya. The clinic has an average total annual attendance of 3,384 patients, with 16.6% comprising PLWE [3].

### Participants

Three hundred consecutive subjects (150 PLWE and 150 accompanying healthy normal controls), who satisfied the inclusion criteria and gave consent were recruited for the study. The PLWE were considered eligible for inclusion if they had been on antiepileptic drug (AED) treatment for duration of at least 2 years and were ≥ 18 years of age. For the normal controls (NC’s) eligibility was if they had no history of ever having suffered a chronic illness and were ≥ 18 years of age. For both groups eligibility for the World Health Organization Quality of Life questionnaire (WHOQOL-BREF) was if they scored ≥ 22 on the Mini-Mental State Examination (MMSE). The presumption was that the accompanying healthy normal controls shared a similar environment and as such similar day to day social experiences as the PLWE they accompanied to the clinic. This was intentionally done in an effort to try and control for foreseen confounders in explaining any differences in QOL outcome determined between the two groups in all the four domains and most importantly the environmental and social relationships domains, so that it would largely be explained by epilepsy status.

### Design

A cross-sectional, comparative study design was used involving the administration of five instruments.

The self-administered WHOQOL –BREF (26 items) questionnaire used in this study, is a short abbreviated form of the WHOQOL-100, which is a comprehensive measure that assesses respondents’ perception and subjective evaluation of various QOL aspects of their lives. The WHOQOL-100 was developed through a culturally diverse multi-center project involving a standardized protocol. The initial testing of the psychometric properties of the WHOQOL-100 involved a pilot study conducted on 4,834 persons in 15 field centers i.e. at least 300 persons, heterogeneous and representative of sick and well people, per center [21-23]. The WHOQOL-BREF was developed at a later stage on the basis of data from the pilot study of the WHOQOL −100, as well as data from 4 new sites. The generic WHOQOL-BREF (26 items) hence satisfies the key properties of a QOL questionnaire i.e. reliability, psychometric validity, responsive to clinical change and being culturally valid [24].

To provide a broad and comprehensive assessment, 24 items (questions) have been included in the WHOQOL-BREF; one item from each of the 24 facets contained in the WHOQOL-100. In addition, two items (questions) from the overall quality of life and general health facets have been included. The WHOQOL-BREF therefore contains a total of 26 items (questions) which make up the facets. These facets are incorporated within a four domain structure i.e. physical health, psychological, social relationships and environment domains with scores scaled in a positive direction (higher percentage scores denote higher quality of life).

For any new centre not previously involved in either the development or field-testing of the WHOQOL-100, the procedure recommended to field test the WHOQOL-BREF should be identical to that used to initially field test the WHOQOL-100. We have therefore used a sample of 300 participants in the current study.

The Mini-Mental State Examination [25] was administered to all respondents and those scoring < 22 were excluded from the WHOQOL –BREF, as it was self-administered. The WHOQOL-BREF was the only self-administered instrument, it was intended to measure the subjective perspective of the participants.

Respondents’ demographic characteristics data including age, gender, level of education, occupation, income, religion, residence, employment and marital status was collected with the researcher’s designed socio-demographic questionnaire.

Data on clinical features of epilepsy was collected using the researcher’s designed seizure burden and characteristics questionnaire. It contained six questions: 1.When did you have the first seizure? 2. Average number of seizures had over the last one year? 3. What, in your opinion, do you think is the cause of your illness? 4. Do you have a history of head trauma? Possible responses for this question were: a) Yes. b) No. If yes, when? 5. Is there any one in your family with a similar illness? Possible responses to this question were a) Yes b) No c) Unknown. If yes, who? a) Other sibling b) Parent c) Grandparent d) Cousin e) Uncle/Auntie f) Others (specify) 6. Type of epilepsy? (From the records): The classification used was from the commission on classification and terminology of the International League Against Epilepsy a) Partial seizures b) Generalized seizures c) Unclassified seizures . Scoring of seizure burden was according to Engel system [26] that scores seizure frequency and disability on a scale ranging from 0–12.

Using the researcher’s designed drug and treatment profile questionnaire data on type of antiepileptic drug (AED) used, duration of treatment (Confirmed from the records), compliance and any associated reasons for non-compliance was collected among PLWE. Additional information on alternative therapeutic approach and patients’ opinion on the effect of medication in their illness was also collected.

### Procedure

The study was carried out at the K.N.H neurology clinic between October 2006 and February 2007. Ethical review was conducted by the Kenyatta National Hospital Ethics Review Committee and approval sought and granted from the Department of Psychiatry at the University of Nairobi. A signed informed consent was obtained from all the subjects involved in the study. The MMSE was then administered to all consenting subjects and those with a MMSE score of < 22 were excluded from the WHOQOL-BREF. Data was then collected using WHOQOL-BREF; the researcher’s designed seizure burden and characteristics, treatment profile, and socio-demographic questionnaires. No names or identifying information were indicated on the questionnaires, and all subjects were assured of confidentiality.

### Data storage and analysis

Collected data was cleaned and stored in a Microsoft Excel database and analyzed using SPSS version 12.0. Descriptive statistics were used to compute means and standard deviations for numerical variables as well as frequencies for nominal and ordinal variables. Significance of association between various variables and QOL was tested using the Chi square test statistic (x^2^). Inferential statistics applied included an independent samples t-test for the hypothesis and in comparing numerical socio-demographic variables. Analysis of variance (ANOVA) was used in comparing mean QOL scores and a stepwise (forward) regression analysis to determine variations in mean QOL as explained by the joint predictive power of the variables. A p < 0.05 was considered statistically significant.

## Results

Three hundred subjects (150 PLWE and 150 accompanying healthy NC’s), participated in the study. As shown in Table 1 below, there was no statistically significant (p > 0.05) difference between the two groups in age, religion, area of residence, household size, children had and gender. The PLWE however showed a statistically significantly lower level of education (p < 0.001), annual income (t = −4.552, p <0.001), and MMSE score (t = −5.212, p < 0.001) as compared to the NC’s. They also had unskilled employment (p = 0.041), with the majority of them being unemployed (p < 0.001) and unmarried (p < 0.001), as compared to the NCs.

**Table 1:** Distribution of socio-demographic variables by epilepsy status

**Variable**	**PLWE (%)**	**Total (%)**	**X**^**2**^	**P value**
**Gender**				
Male	78 (52.0)	139 (46.3)		
Female	72 (48.0)	161 (53.7)	3.874	0.064
**Residence**				
Urban	75 (50.0)	154 (51.3)		
Rural	75 (50.0)	146 (48.7)	0.213	0.729
**Religion**				
Protestant	92 (61.3)	189 (63.0)		
Catholic	56 (37.3)	108 (36.0)		
Others	2 (1.4)	3 (1.0)	0.614	0.736
**Level of education**				
No formal	10 (6.7)	11 (3.7)		
Primary	53 (35.3)	79 (26.3)		
Secondary	61 (40.7)	113 (37.7)		
Tertiary	18 (12.0)	81 (27.0)		
University	8 (5.3)	16 (5.3)	44.132	<0.001*
**Marital status**				
Ever married	69 (46.0)	171 (57.0)		
Never married	81 (54.0)	129 (43.0)	14.81	<0.001*
**Employment status**				
Employed	62 (41.3)	174 (58.0)		
Unemployed	88 (58.7)	126 (42.0)	34.209	<0.001*
**Types of employment**				
Skilled	20 (32.3)	74 (42.5)		
Unskilled	42 (67.7)	100 (57.5)	4.157	0.041*

*Statistically significant.

### PLWE

Among the 150 PLWE who participated in this study, 74.7% had generalized seizures, 23.3% partial, whereas only 2% had unclassified seizures. A total of 37.3% of the PLWE reported using alternative modes of therapy. The commonly used alternative modes of therapy included prayers (21.3%), herbs (12.7%), and witchcraft (2.7%). Majority, (92.7%), of the PLWE in this study reported improvement as a result of AED treatment with 83.3% of them even reporting expectations of being cured. Over half (53.4%) of the unemployed PLWE in this study blamed their illness (epilepsy) as the reason for their unemployment while 50.7% of the PLWE reported not knowing the cause of their illness.

The factors that were found to be statistically significantly associated with a higher seizure burden included use of poly AED’s therapy (x^2^ = 19.406, p < 0.001), being unmarried (x^2^ = 8.593, p = 0.035), use of alternative therapy (x^2^ = 8.585, p = 0.035), low annual income (f = 3.161, p = 0.027), low MMSE scores (f = 4.029, p = 0.009), a longer duration of illness (f = 3.392, p = 0.020), a past history of head injury (f = 3.117, p = 0.026) and an earlier age at onset of epilepsy (f = 5.633, p = 0.001).

### QOL

Out of the 300 participants in this study, 285 (150 NC’s and 135 PLWE) satisfied the inclusion criteria (MMSE score of ≥ 22) for the self- administered WHOQOL-BREF questionnaire.

The mean QOL among PLWE (49.90%), t = −17.694, p < 0.01, at K.N.H was statistically significantly lower than that of the NC’s (77.60%), t = −18.298, p < 0.01, accompanying them and also statistically significantly impaired, t = −18.298, p < 0.01, as compared to the hypothesized mean of 75^±^2.5% [27].

**Figure 1:**
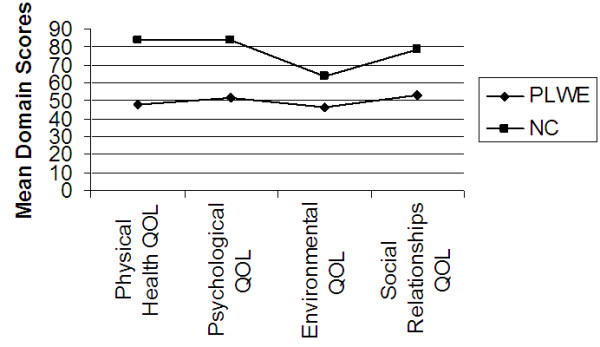
**Mean domain scores as determinants of mean QOL.** X axis represents the four QOL domains using the WHOQOL-BREF. Y axis represents the mean domain scores (%).

**Figure 2:**
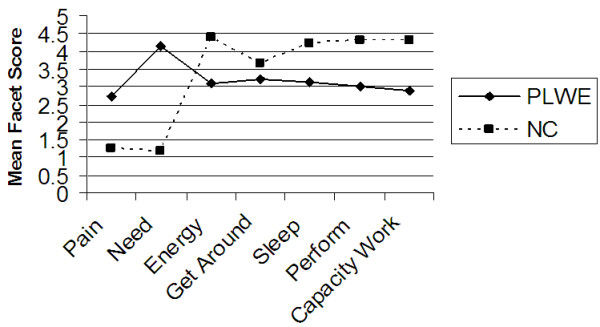
**Facets determining the physical health QOL domain.** X axis represents the seven facets that comprise the physical health QOL domain. Y axis represents the mean facet score (0 to 5).

**Figure 3:**
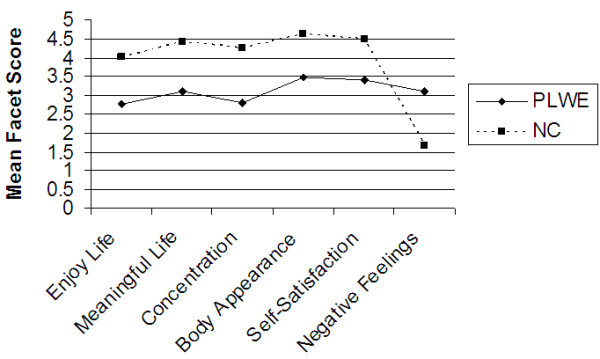
**Facets determining the psychological QOL domain.** X axis represents the six facets that comprise the psychological QOL domain. Y axis represents the mean facet score (0 to 5).

**Figure 4:**
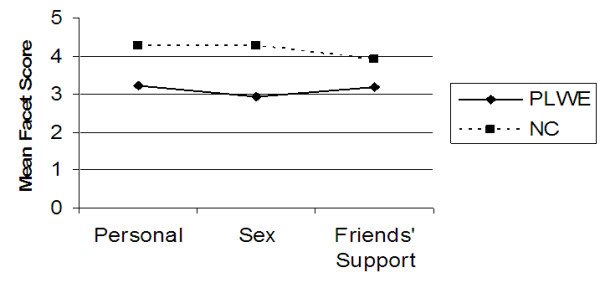
**Facets determining the social relationships QOL domain.** X axis represents the three facets that comprise the social relationships QOL domain. Y axis represents the mean facet score (0 to 5).

**Figure 5:**
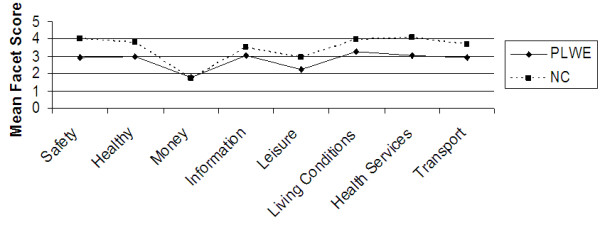
**Facets determining the environmental QOL domain.** X axis represents the eight facets that comprise the environmental QOL domain. Y axis represents the mean facet score (0 to 5).

Further analysis of the data was carried out to determine the difference in mean domain and facet QOL scores between PLWE and NC’s using the independent samples test (t-test) at 99% confidence interval. Figures 1, 2, 3, 4, 5 illustrate these findings.

As shown in Figure 1, the mean QOL scores for each of the four domains used in the WHOQOL-BREF i.e. physical health QOL (t = − 19.859), psychological QOL (t = −18.698), social relationships QOL (t = −9.934) and environmental QOL (t = −9.934) were all statistically significantly (p < 0.01) lower for PLWE as compared to the NC’s.

As illustrated in Figures 2, 3, 4, 5 above, all the mean facet QOL scores were also statistically significantly (p < 0.01) lower in PLWE as compared to the NC’s, apart from the subjective evaluation on financial resources. Both groups reported not having enough money to meet their needs, t = 0.489, p = 0.625.

As shown in Table 2 below, the factors statistically significantly associated with impairment of QOL among PLWE were a low level of education (p < 0.001), higher seizure burden (p < 0.001), low annual income (p = 0.007), unemployment (p = 0.004), unskilled employment (p < 0.001), and rural residence (p = 0.009). Additionally, those PLWE who reported financial difficulties as the reason for non-compliance to treatment (p = 0.037) and those who blamed their illness (epilepsy) as the cause of their unemployment (p < 0.001) also showed statistically significantly impaired QOL.

**Table 2:** Mean QOL and various variables of PLWE

**Variables**	**N**	**Mean QOL**	**F value**	**P value**
**Residence**				
Urban	71	53.29		
Rural	64	46.14	7.079 **ₓₓ**	0.009*
**Level of education**				
No formal	4	48.62		
Primary	45	42.60		
Secondary	58	50.15		
Tertiary	20	61.10	23.752 **ₓ**	<0.001*
University	8	61.78	7.022 **ₓₓ**	<0.001*
**Employment status**				
Employed	60	54.29		
Unemployed	75	46.39	8.656 **ₓₓ**	0.004*
**Type of employment**				
Skilled	19	65.78		
Unskilled	41	48.96	22.290 **ₓₓ**	<0.001*
**Income / month (Kshs)**				
None	75	46.39		
1-22999	8	47.15		
23000 – 59999	15	49.48		
60000 – 119999	12	50.77		
120000 – 179999	13	60.53		
180000 – 359999	8	58.90	16.700 **ₓ**	<0.001*
360000 or more	4	67.62	3.144 **ₓₓ**	0.007*
**Seizure Burden**				
No seizures last one year	37	54.28		
1-3 seizures last one year	47	54.26		
4-11 seizures last one year	19	46.05	17.880 **ₓ**	<0.001*
> 12 seizures last one year	32	40.72	6.789 **ₓₓ**	<0.001*
**Reasons for non-compliance**				
Side effects	5	59.70		
Financial difficulties	30	47.48		
Forgot to take	9	62.72	2.235 **ₓ**	0.142
Others	7	59.07	3.057 **ₓₓ**	0.037*
**Reason for unemployment**				
Due to disease	37	39.45		
Not due to disease	12	47.39		
Retired	4	49.00	20.385 **ₓ**	<0.001*
Student	22	57.04	7.100 **ₓₓ**	<0.001*

**ₓ** Value for linearity.

**ₓₓ** Value between groups.

* Statistically significant.

There was no statistically significant (p > 0.05) relationship between mean QOL of PLWE and gender, marital status, age, children had, household size, mode and specific type of drug therapy, seizure type, age at onset of epilepsy, duration of illness or duration of treatment.

In order to determine which variables to include in a regression analysis, all independent variables of PLWE were correlated with the dependent variable, mean QOL, using bivariate (Pearson) correlation. The regression model used then showed that, 11.6% of variation in mean QOL was explained by level of education, 8.1%, average annual seizures, 5.0%, reason for unemployment, 4.5% average annual income, and 2.3%, by type of employment. These variables therefore explained 31.5% of the total variations in mean QOL. The residuals plots showed that data met the assumptions of linearity, homoscedasticity and normality in the regression model used.

## Discussion

### Quality of life

The mean QOL of PLWE (49.90%) in this study was significantly lower than that of the NC’s (77.60%) and was also significantly impaired as compared to the hypothesized mean (75%). This is similar to the findings in a 2004 Tunisian study [4] that compared PLWE with a general reference population and also similar to the findings in a Malaysian pilot study of the WHOQOL-100 [19]. The mean QOL of PLWE in this study is slightly higher but comparable to that reported (44%) among the Dutch PLWE [8] in a 2001 study. The implication of this finding is that the approach to the management of PLWE in Kenya should not only focus, as is traditionally done, on seizure control but instead adopt a holistic approach that also incorporates their psychological, social and environmental needs. In the present study, the PLWE were dissatisfied with their access to health services, further reflecting the need to improve the provision of accessible health services in our nation and the role of the health service provider in reducing the treatment gap. This gap is however not unique to Kenya, as in 2001 the World Health Organization (WHO) and the International League Against Epilepsy (ILAE) estimated that nearly 80% of the PLWE in the developing countries are not on treatment [5]. Establishment of accessible, well integrated health services that implement a multi-disciplinary approach to the care of PLWE in our country, with advocacy aimed at involving the patients, their families and significant others would be invaluable in reducing the treatment gap and improving their QOL.

Unlike the NC’s , the PLWE needed treatment to function in their daily lives, felt that physical pain prevented them from achieving what they wanted in life and were not at all satisfied with their capacity for work. They also subjectively reported a diminished ability to concentrate, suffered frequent negative feelings and felt that their life was not meaningful. Other studies [8,14-16,19,28] have reported similar findings.

In Kenya, like in most African settings, ‘pain’ usually refers to intangible emotions such as despair, hopelessness, helplessness, and dysphoria. This finding probably reflects that some signs of depression cannot be discounted in this population of PLWE. It is therefore not surprising that they expressed dissatisfaction with their sex life, personal relationships, support from their friends, and felt unsafe, while in a similar environment to that of the NC’s. Early psychiatric assessment, with psycho-education, socio-occupational evaluation and skills training would probably improve their outcome. Such interventions have been implemented among PLWE elsewhere [10] with favourable outcome and improvement in QOL.

### Level of education

In this study, the level of education attained was the most important factor explaining variations in QOL. The findings showed that the PLWE had attained a significantly lower level of education as compared to the NC’s. This probably reflects the fact that with a good education the PLWE would get better earning opportunities through skilled employment, making it easier to afford and access health services, choose where to reside, instead of blaming their illness as the reason for their unemployment. The results therefore suggest the need for the enforcement of measures aimed at creating better educational opportunities for PLWE by eliminating the associated stigma and prejudice towards them in learning institutions. In fact an earlier study [29] had predicted the deterioration of a classroom environment with the addition of a pupil living with epilepsy.

### Seizure burden

In this study increasing seizure frequency was the only clinical variable that was found to be significantly associated with QOL impairment among the PLWE in all the domains. Other studies [4,7,11,19] have reported similar findings, with seizure frequency being reported as an inverse predictor of QOL among PLWE. Mativo, in a 2004 study [3] carried out in the same locality, found that among other factors poly AED’s therapy (30%) and the use of alternative therapy (22%) were associated with poor control of epilepsy. The use of poly AED’s therapy (54%) and alternative therapy (37.3%), as reported in the current study, has therefore almost doubled since 2004. This is worrying given that these are among the factors significantly associated with higher seizure frequency and impaired QOL among PLWE. Literature [30,31] has suggested that a thorough epilepsy classification is the first and most important step, followed by using the recommended optimal dose of mono AED therapy for that specific type before introducing a second AED, as treatment is type specific. Elsewhere [32], the impact of surgical intervention in correlation with seizure control on QOL of PLWE has been reported as positive in all ages.

Early age at onset of epilepsy or childhood onset epilepsy was another factor significantly associated with higher seizure frequency that also ultimately impaired QOL among PLWE. Reported outcomes of childhood onset epilepsy [33] include stigmatization, unemployment, and can also be considered as a marker for adverse outcome on QOL. Other factors significantly associated with higher seizure frequency included a past history of head injury, longer duration of illness, low annual income, low MMSE score and being unmarried.

### Unemployment

A much higher unemployment rate (58.7%) was found among PLWE as compared to the NC’s (25.3%) in the current study, and also to that reported (25%) by Julie et al. in a 2003 study involving respondents from 10 European countries [12]. All these rates are higher than the overall national unemployment rate (12.7%) in Kenya [34]. Notable though, was that over half (53.4%) of these unemployed PLWE in this study blamed their illness (epilepsy) as the cause of their unemployment. This level, though lower than that reported by Hela et al. (62%) in a 2002 Tunisian study [4], is still significant. As literature [29,35] has shown, this finding probably reflects a population of PLWE that has undergone recurrent and sustained levels of objective stigma until they have accepted the situation, internalized the damaging opinions as if they were their own and developed subjective stigma. Muinga [2] in a study carried out at the same locality also reported that psychopathology in PLWE was positively correlated with unemployment while another recent local study [3] reported that up to 63% of the unemployed PLWE were poorly controlled.

### Unskilled employment

In this study 42 (28%) of the PLWE had unskilled employment. This population of the PLWE expressed dissatisfaction with their capacity for work, ability to concentrate, perform daily activities, and with the support they got from their friends. Studies [14-16] have shown that the unfavourable public attitude towards PLWE and the negative expectations from their workmates, subsequently leads to increased ‘secrecy’ about their condition, further reflecting the role of stigma in QOL impairment. Literature [1] has also shown that, among PLWE, an imbalance between their expectations and reality may be more menacing than the illness itself. There is therefore a need for de-stigmatization of epilepsy through mass-media and various other forums, with the aim of creating awareness, improving employment opportunities and addressing employment related stigma among PLWE in our nation.

### Income

The preceding discussion on the low level of skilled employment, lower level of education attained, and the high level of unemployment among PLWE in this study, is a plausible explanation for their lower annual income as compared to the NC’s. In this study, low socio-economic status and more specifically low annual income among the PLWE, was associated with significantly poorer QOL. It was also noted that this association showed significant linearity i.e. as their income increased their mean QOL improved.

### Residence

Despite showing no significant difference in residence between the PLWE and the NC’s, those PLWE from the rural community subjectively reported dissatisfaction with their access to health services. They also felt their life was meaningless, reported dissatisfaction with their sex life, ability to get around and availability of information they needed in their day-to-day life. This group, of PLWE from a rural residence, was additionally dissatisfied with their sleep, ability to perform daily activities and subjectively suffered more frequent negative feelings than their urban counterparts. As mentioned earlier, the inaccessibility of health services interferes with optimal care for PLWE, and contributes significantly to the treatment gap [5] among PLWE in developing countries.

### Limitations

A major limitation is the fact that a self-administered questionnaire (WHOQOL-BREF) was used to collect information on QOL, yet it had not been translated to the national language Kiswahili. Despite the fact that none of the subjects reported any problem with the comprehension of the WHOQOL-BREF, a translation would have eased their interpretation enabling more subjectivity to be captured.

## Conclusions

This study has demonstrated a compromised QOL among PLWE as compared to healthy NC’s attending a neurology clinic in a national and major referral Kenyan hospital. Factors significantly associated with impairment of QOL among the PLWE included a low level of education, higher seizure burden, low annual income, unemployment, unskilled employment, and living in a rural residence. It is recommended that advocacy of services geared towards de-stigmatization and enforcement of measures aimed at improving educational opportunities for the PLWE in Kenya be instituted. The need for early empowerment of the PLWE through involvement of the family and significant others would also be useful in improving the QOL of PLWE in our country. Invaluable also, is the establishment of a comprehensive epilepsy management program to coordinate service delivery using a multidisciplinary approach, which would include and incorporate the role of surgical intervention, in the management of PLWE in Kenya. Improvements in the availability, accessibility and affordability of AED’s especially in the rural areas would also drastically reduce the treatment gap and subsequently improve QOL of PLWE in this region.

## Abbreviations

AED: Antiepileptic drug; ANOVA: Analysis of variance; ILAE: International league against epilepsy; KNH: Kenyatta national hospital; MMSE: Mini-mental state examination; NC: Normal controls; PLWE: People living with epilepsy; QOL: Quality of life; SF-36: Short form survey- 36 questionnaire; WHO: World health organization; WHOQOL-BREF: World health organization quality of life- BREF questionnaire; WHOQOL-100: World health organization quality of life- 100 questionnaire.

## Competing interests

The authors declare that they have no competing interests.

## Authors’ contributions

All authors were involved in the conceptualisation of the study, participated in its design, and performed statistical analysis of the data. DMK and JMM were involved in supervision of data collection. WDCK carried out the acquisition of data and was involved in preparation of the final manuscript. All authors read and approved the final manuscript.

## References

[B1] Theo PBMS Marieke FR Bert PA Quality of life in Epilepsy: Multidimensional profile and underlying latent dimensions J Epilepsy 1998 11 84 97 10.1016/S0896-6974(97)00141-2

[B2] Muinga EG Psychiatric morbidity in epileptics as seen in a neurology outpatient clinic, KNH 1986 Nairobi Kenya: M. Med Dissertation

[B3] Mativo PM Factors associated with poor control of epilepsy at KNH, Nairobi, Kenya 2004 M. Med Dissertation: Adult Neurology Clinic

[B4] Hela M Amel M Bechir Z Health related quality of life of people with epilepsy compared with a general Reference population. A Tunisian study Epilepsia 2004 45 838 843 10.1111/j.0013-9580.2004.56903.x 15230710

[B5] WHO Fact sheet No. 168 Epilepsy: an Historical overview 1998 WHO/OMS

[B6] Kobau R Health-related quality of life among adults with epilepsy, BRFSS, Texas J Am Med Assoc 1998 47 135 140

[B7] Liou HH Chen RC Chen CC Health related quality of life in adult patients with epilepsy compared with a general reference population in Taiwan Epilepsy Res 2005 64 151 159 10.1016/j.eplepsyres.2005.03.006 15935621

[B8] Theo PBMS Marieke FR Bert PA Social functioning, psychological Functioning and Quality of Life in epilepsy Epilepsia 2001 42 1160 1168 11580765 10.1046/j.1528-1157.2001.37000.x

[B9] Choi-kwon S Chung C Kim H Factors affecting the quality of life in patients with epilepsy in Seoul, South Korea Acta Neurol Scand 2003 108 428 434 10.1046/j.1600-0404.2003.00151.x 14616296

[B10] Gunter MJ Brixner D Von Worley A Impact of a Seizure disorder disease management program on patient reported quality of life DisManag 2004 4 333 347 10.1089/dis.2004.7.333 15671790

[B11] Leidy NK Elixhauser A Vickrey B Seizure frequency and the health related quality of life of adults with epilepsy. American Academy of Neurology Neurology 1999 53 162 166 10.1212/WNL.53.1.162 10408553

[B12] Julie D Gus AB Ann J Cross-Cultural Differences in levels of knowledge about epilepsy Epilepsia 2003 44 115 123 10.1046/j.1528-1157.2003.34402.x 12581238

[B13] Pedroso de Souza EA Priscila C Barioni S A psychosocial view of anxiety and depression in epilepsy Epilepsy & Behaviour 2006 8 232 238 10.1016/j.yebeh.2005.10.011 16356782

[B14] Gus AB Ann J Joanne G Quality of Life of people with Epilepsy in Iran, the Gulf, and the near east Epilepsia 2005 46 132 140 10.1111/j.0013-9580.2005.20704.x 15660779

[B15] Ann J Joanne G Carol G Public knowledge, Private Grief: A study of public attitude to epilepsy in the United Kingdom and Implications for Stigma Epilepsia 2004 45 1405 10.1111/j.0013-9580.2004.02904.x 15509242

[B16] Kelly P Is perceived stigma related to Quality of life in Individuals with Epilepsy? Department of Experimental Psychology 2004 MSC Project: University of Bristol

[B17] Amir M Roziner I Knoll A Self-efficacy and Social support as mediators in the relation between disease severity and quality of life in patients with epilepsy Epilepsia 1999 40 216 224 10.1111/j.1528-1157.1999.tb02078.x 9952270

[B18] Jenna M New study reveals significant link between Depression and anxiety and decrease in QOL in epilepsy patients Epilepsy & Behaviour 2005 6 563 569 10.1016/j.yebeh.2005.02.017 23785660 PMC3683288

[B19] Hasanah CI Razali MS The pilot study of WHOQOL-100 (MALAY VERSION) Malays J Med Sci 1999 6 21 25 22589685 PMC3329746

[B20] Gabriel MR David LS Peter R Health-related quality of life in childhood epilepsy: Moving beyond 'seizure control with minimal adverse effects' Health Qual Life Outcomes 2003 1 36 10.1186/1477-7525-1-36 14498989 PMC201010

[B21] Special Report Study protocol for the World Health Organization project to develop a quality of life assessment instrument (WHOQOL) Qual Life Res 1993 2 153 159 8518769

[B22] Mick P Willem K The WHOQOL Group (1994a,1994b, in preparation): The WHOQOL Assessment (WHOQOL). Development and general psychometric properties Soc Sci Med 1998 46 1569 1585 10.1016/S0277-9536(98)00009-4 9672396

[B23] WHOQOL Group The World Health Organization Quality of life assessment (WHOQOL): Development and general psychometric properties Soc Sci Med 1998 46 1569 1585 10.1016/S0277-9536(98)00009-4 9672396

[B24] Andrea B The Basics of QOL: Study instruments or Practice-Oriented Data? MASCC/ISOO: 17th International Symposium 2005 Geneva, Switzerland: Supportive Care in Cancer June 30- July 2nd

[B25] Sadock BJ Sadock VA Kaplan and Sadock's Synopsis of Psychiatry. Behavioural Sciences/ Clinical Psychiatry 2003 9 Lippincott Williams & Wilkins 321

[B26] Engel J Jr Ness VR Ojemann LM Engel JJr Outcome with respect to epileptic Seizures Surgical treatment of the epilepsies 1993 New York: Raven press 609 622

[B27] Cummins RA On the trail of the gold standard for subjective well-being Soc Indic Res 1995 35 179 200 10.1007/BF01079026

[B28] Baker GA Jacoby A Buck D Quality of life of people with epilepsy: a European Study Epilepsia 1997 38 353 362 10.1111/j.1528-1157.1997.tb01128.x 9070599

[B29] Baumann RJ Wilson JF Wiese HJ Kentuckians’ Attitude Toward children with Epilepsy. Epilepsy Education and prevention activities Database. Chronic Disease prevention Database Epilepsia 1995 36 1003 1008 10.1111/j.1528-1157.1995.tb00959.x 7555950

[B30] Carrieri PB Provitera V Iacovitti B Mood disorders in epilepsy Acta Neurol Napoli 1993 15 62 67 8456597

[B31] Philip NP Walter F Francesco P The importance of drug interactions in epilepsy therapy Epilepsia 2002 43 365 10.1046/j.1528-1157.2002.13001.x 11952767

[B32] Berto P Quality of Life in Patients with epilepsy and Impact of Treatments PharmacoEconomics 2002 20 1039 1059 10.2165/00019053-200220150-00002 12456200

[B33] Matti S Adults taking epileptic medications more likely to be unemployed Epilepsia 1997 38 708 10.1111/j.1528-1157.1997.tb01241.x 9186254

[B34] Kenya National Bureau of Statistics Kenya Integrated Household Budget Survey (2005/2006) 2007 Nairobi: Government Printer

[B35] Temkin O History of Epilepsy: The falling sickness 1945 Baltimore, USA: John Hopkins press

